# Identifying the determinants of COVID-19 preventative behaviors and vaccine intentions among South Carolina residents

**DOI:** 10.1371/journal.pone.0256178

**Published:** 2021-08-25

**Authors:** Justin Travis, Scott Harris, Tina Fadel, Ginny Webb

**Affiliations:** 1 Department of Psychology, University of South Carolina Upstate, Spartanburg, SC, United States of America; 2 Department of History, Political Science, Philosophy, & American Studies, University of South Carolina Upstate, Spartanburg, SC, United States of America; 3 Division of Natural Sciences and Engineering, University of South Carolina Upstate, Spartanburg, SC, United States of America; University of Haifa, ISRAEL

## Abstract

Coronavirus disease 2019 (COVID-19) has presented a global pandemic in 2020 and 2021, and has therefore spurred a flurry of research, whether related directly to the disease and its treatment or regarding its spread, containment, and effect on everyday lives. In particular, two pressing streams of research have investigated antecedents to COVID-19 preventative behaviors and vaccination intentions. This nascent research has led to many interesting and practically important findings, however, there remains many segmented, compartmentalized studies that address topics that, while certainly generative and meaningful, may not provide a full lens to possible antecedents. The current study takes an interdisciplinary approach that investigates commonly studied variables from biology and public health, political science, and psychology as they relate to COVID-19 preventative behaviors and vaccine intentions in a stratified sample of South Carolina residents (*N* = 1695). Results from correlations and multiple regression substantiated the findings of many previous studies, however, it was found that, when controlling for shared variance among predictors via relative weights analysis, COVID-19 knowledge, trust in science, age, and Trump approval were the strongest predictors of preventative behaviors. Alternatively, trust in science, gender, age, and conservatism were the strongest predictors of vaccine intentions. Understanding the variables that contribute to the practice of preventative behaviors and vaccine intentions can be used by public health officials to better target and tailor their educational campaign in the community.

## Introduction

Coronaviruses are a family of viruses responsible for many respiratory diseases, ranging from mild to severe. [1]. Coronavirus disease 2019 (COVID-19) is a respiratory disease caused by the severe acute respiratory syndrome coronavirus 2 (SARS-CoV-2) that ranges in severity from mild to severe with symptoms including fever, cough, shortness of breath, and fatigue [2]. The likelihood of severe disease or death from COVID-19 significantly increases with age as well as with certain comorbidities including diabetes, heart disease, lung disease, obesity, and kidney disease [3]. Transmission of SARS-CoV-2 occurs primarily through respiratory droplets from close contact. It is also important to note that up to 50% of disease transmission occurs from individuals with presymptomatic or asymptomatic infections [2].

As of March 19, 2021, COVID-19 has caused over 29 million cases and over 536,000 deaths in the United States alone [3]. The Centers for Disease Control and Prevention (CDC) and World Health Organization (WHO) have provided guidelines to limit the spread of COVID-19 in an effort to decrease incidence rates, lower hospital burden, and prevent deaths. The most effective preventative measures are wearing a mask, social distancing by staying six feet away from others, and avoiding crowds or gatherings [3, 4]. Due to the ease of respiratory droplet transmission, combined with the knowledge that transmission can occur in asymptomatic individuals, it is vitally important that individuals follow the preventative guidelines in order to maintain low levels of community spread of COVID-19. Although these preventative measures are backed by peer reviewed scientific studies and have been recommended and/or mandated to the public by government or public health officials, there have been varying levels of compliance.

In addition to these important preventative behaviors the other key to decreasing COVID-19 transmission is vaccination. Vaccine development began immediately at the beginning of the pandemic. Reaching herd immunity through vaccination is key to ending the pandemic and providing protection at both the individual and community levels. Two vaccines received Emergency Use Authorization (EUA) from the US Food and Drug Administration in December of 2020, however, there has been hesitancy in receiving the vaccine, with approximately 70% of the population expressing willingness to receive the vaccine [5, 6]. There are several reasons contributing to vaccine hesitancy including concerns over the safety of the vaccine, misinformation, and belief that the disease is only mild [7]. Given, the public health consensus, a crucially important task for researchers across disciplines is to assess what is driving individual differences in engaging in preventative behaviors as well as vaccine acceptance.

Previous studies have begun examining factors related to COVID-19 preventative behavior compliance. One factor that has been found to influence compliance to these behaviors is general knowledge about the virus and disease [8–10]. An individual’s knowledge related to the pandemic and/or COVID-19 has been found to be positively related to preventative behaviors [8, 11]. In addition to general scientific knowledge, some demographic characteristics (e.g., age, gender and SES) also predict acceptance and practice of preventative behaviors [8, 9, 12]. Mask wearing has been shown to be influenced by both age and gender with older individuals and females being more likely to wear a mask [9, 13, 14]. Also, those in urban locations are more likely to adhere to mask wearing compared to individuals in rural geographies [13]. Many of the same variables have been shown to contribute to vaccine acceptance rates. Increased age and knowledge about the disease have both been shown to affect vaccine acceptance [15]. There are conflicting reports on the role of gender in vaccine acceptance with Lazarus *et al* [5]. reporting females as being more likely while Kreps *et al*. [16] reported females to be less likely to accept a vaccine.

Previous studies have measured the population’s trust in science and more recently some studies have started examining the beliefs and practices of the population in relation to COVID-19. This nascent research has documented the role of various psychological constructs in relation to the adoption of widely supported preventative behaviors. Many authors have taken an approach focusing on potential hurdles to individuals’ adoption of preventative behaviors, such as beliefs in conspiracy theories [17, 18], trust in science and/or scientists [12, 19], trust in politics [20], and need for cognition [21].

Moreover, such variables have also been found to predict vaccine hesitancy and/or intentions to receive a COVID-19 vaccine (e.g. [22, 23]). Collectively, these findings suggest that multiple psychological variables (e.g., trust in science and need for cognition) may be predictive of important criteria involved in the spread, containment, and otherwise future trajectory of COVID-19. In studying the antecedents of preventative behaviors, however, it is important that demographic, social, and psychological variables are studied alongside what has become an increasingly charged area of individual differences–political attitudes and beliefs.

Studies have shown that political beliefs/identity predict COVID-19 preventative behaviors [24–26]. Several studies find partisan gaps in commitments to preventative measures, with Republicans less willing to take such measures than Democrats [24–26]. As to willingness to take a COVID-19 vaccine, polling and at least one study have suggested Democrats were more willing to take a vaccine for the virus than Republicans [16, 27].

A missing link in evaluating the partisan gap that exists in this public health crisis is a more complete understanding of the causal mechanism driving the partisan gap. Certainly, demographics play some role, as males and rural Americans are both disproportionately Republican and have been found to be less willing to engage in preventative behavior [13, 14, 28–30]. On the other hand, females and Black citizens, both disproportionately Democratic groups, have been found in one study to be *less* likely to take the vaccine, at least prior to approval [16]. Further, older Americans skew Republican [31] but have been found to both be more likely to engage in preventative measures [9, 13] and more willing to take the vaccine [16].

Thus, it seems demographics alone cannot fully explain compliance with COVID-19 public health guidance. One mechanism that could be shaping this compliance is partisan messaging, with some evidence that partisan leaders can shape public health behavior [32–34]. [32–34] However, other studies find partisan elites have limited power to influence the public’s response to the COVID-19 crisis [25, 35, 36]. A different potential explanation for these disparate findings is that pure conservative ideology, rather than elite-cueing, drives the partisan gap in compliance with public health guidance [26, 37]. This theory suggests a lack of trust in government, or simply anti-government opinions, among conservatives renders them less likely to comply with guidance from government officials. Overall, a review of the findings on the link between political views and COVID-19 behavior suggests that additional testing is needed to determine the relative weight of several potential important political and demographic attributes [8, 12, 19].

Ultimately, we know that there are complex relationships between many of the factors that affect an individual’s compliance to preventative behaviors. For example, Fridman *et al*. [11] demonstrated that COVID knowledge is associated with what information sources are trusted by the individual and the knowledge level then affects the likelihood of preventative behavior compliance. Similarly, Dohle *et al*. [12] found that multiple factors are associated with preventative behaviors including demographic factors, with women and older individuals more likely to engage in such behaviors. But they also found associations with various social behaviors, including trust in science and trust in government. Thus, it is important to begin to examine the complexity of the interplay of varying factors and how their relationships affect preventative behaviors.

Studies have shown that a decrease in preventative behaviors is correlated to increase community spread of COVID-19. As disease levels increase in the community, hospitals become overburdened which may result in rationing of care. In order to keep hospitals fully functioning and decrease the number of excess deaths, it is vital for individuals to practice the appropriate preventative behaviors. It is important to understand the factors influencing compliance to preventative behaviors so that public health officials can effectively communicate and educate the community about the necessity of practicing these behaviors.

In this study, we take a broad and holistic approach to examining the individual factors that influence one’s compliance to preventative behaviors as well as vaccine acceptance. We specifically examined individuals from the state of South Carolina (SC). SC does not have a state-wide mask mandate, but some individual counties or cities have implemented their own mask-mandates. Thus, residents of SC are likely to show higher variation in prevention behaviors, especially when it comes to wearing a mask, compared to states with state-wide, uniform mandates on prevention behaviors. SC has had high incidence rates of COVID-19 with surges occurring in the Summer of 2020 and the winter of 2020–21. There has been much divide among individuals within SC about the importance and individual duties of complying with COVID-19 preventative behaviors. Considering the results of nascent literature on individual-level compliance with COVID-19 guidelines and vaccine hesitancy/intentions, we aimed to determine which variables are most predictive of COVID-19 preventative behaviors and vaccine intentions.

## Materials and methods

### Participants and procedure

The protocol and procedures used in our data collection were approved by the Institutional Review Board (IRB) at the University of South Carolina. All participants were required to provide informed consent in digital form prior to participation in our study. In order to obtain a representative sample of South Carolina residents, we employed Qualtrics to solicit a stratified sample that drew from each county in South Carolina. Qualtrics’ proprietary procedures were employed, consisting largely of online recruitment and e-mail solicitation, with the goal of representing the state geographically. Individuals that met the criteria were invited to take the survey by Qualtrics via e-mail. Criteria for participation was that the individual was a resident of South Carolina and 18 years of age or older. In total, 1707 residents provided viable data for the online survey.

Once they agreed to participate, participants were taken to the online survey, hosted by Qualtrics, and completed a battery of questionnaires that lasted approximately 25 minutes. Survey responses were collected from October 15^th^ until November 8^th^. After taking part in the survey, participants were thanked and compensated by Qualtrics per their agreement with Qualtrics.

Participants were mostly female (74.1%), white (75%), with some college education (25.5%), and averaged 43 years of age. Our sample approximated the latest Census estimates in most demographics, except Gender (estimated 52% female) [38]. However, gender-weighting to match this population does not substantially change any of our key results. The counties with the most participants, such as Greenville (11.3%), Charleston (9.6%), Horry (8.9%), and Richland (7.2%), are also the largest counties by population in South Carolina [39].

### Measures

#### COVID-19 knowledge test

To assess COVID-19 knowledge, participants were asked 11 questions covering information on the transmission, symptoms, and treatment of COVID-19. Questions were adopted from previous research [8, 9]. Responses were scored as correct (1) or incorrect (0) and summated across the 11 items. Scores ranged from 1 to 11 (*M* = 9.55, *SD* = 1.67).

#### Close contacts with COVID-19

Subjects were asked to report, using “yes” or “no,” two questions: whether they had a close contact that had a mild case of COVID-19 and whether they had a close contact that died from COVID-19.

#### Need for cognition

Need for cognition refers to individual differences in a person’s proclivity and interest in engaging in effortful, deliberative processing of information (see [40]). Participants answered the International Personality Item Pool’s (IPIP) [41] 10-item Need for Cognition scale, anchored from 1 = *Strongly Disagree* to 7 = *Strongly Agree* (*α* = .85).

#### Trust in science

A 21-item measure of trust in science and scientists was used (see [42]) with sample items including, “Scientists intentionally keep their work secret” and “We can trust science to find the answers that explain the natural world.” Responses were recorded on an agreement scale anchored from 1 = *Strongly Disagree* to 7 = *Strongly Agree* (*α* = .94).

#### 2020 vote/preference

Participants were asked, “If the 2020 election were held today, who would you vote for? If you have already voted, who did you vote for?” Response options were limited to Joe Biden, Donald Trump, “someone else,” and “Will not vote/unsure.”

#### Political ideology

To measure ideology, we used the standard self-reported 7-point scale ranging from “very liberal” to “very conservative.” Higher values reflect more conservative respondents.

#### Party identification

To measure political partisan identification on the customary 7-point scale [43], participants were first asked whether they identify as a Republican, a Democrat, Independent, or something else. Republicans and Democrats were then asked respectively whether they consider themselves “strong” or “weak.” Respondents who did not initially identify with either major party were asked whether they are closer to the Republican Party, Democratic Party, or neither. This method is used to capture “leaners” or “closet partisans” who initially identify as independent but later answer they are closer to one of the two major parties. Prior research has found leaners to behave more like partisans than true independents [44, 45]. The resultant measure is computed as 1 = Strong Democrat, 2 = Weak Democrat, 3 = Democratic Leaner, 4 = Independent, 5 = Republican Leaner, 6 = Weak Republican, 7 = Strong Republican.

#### Trump approval

Trump approval was measured by asking respondents to respond with “approve” or “disapprove” to the question, “Do you approve or disapprove of the way President Trump is doing his job?”

#### Preventative behaviors

Three questions measured participants’ preventative behaviors that aligned with CDC and contemporary medical guidance, "How often do you thoroughly wash your hands with soap and water or use alcohol-based hand sanitizer”, “How often do you wear a mask when entering a public place even if it is not required”, and “How often do you practice social distancing of 6 feet when you are in public?” Response options were on a four-point scale anchored from *never* to *always* (*α* = .70).

#### Vaccine intentions

Participants were asked, “Assuming approval by the FDA (Federal Drug Administration), do you plan to take a vaccine for COVID-19 if and when it becomes available?” Responses were recorded as “yes” or “no.”

#### Demographic control variables

Several demographic variables were measured using self-report including age, gender, race, education level, and county of residence. Additionally, county-level population density was paired based on self-reported county of residence.

## Results

Prior to data analysis, survey responses were first screened for careless responding (see [46]). Although Qualtrics performs their own proprietary checks for data integrity, a conservative assessment of careless responding was conducted whereby data was examined for maximum longstring responses (nine or more consecutive identical responses across multi-item scales). This survey consisted of several measures of disparate constructs with both positive and negatively coded items. Therefore, it was deemed extremely unlikely that an individual would respond to different scales and different items within and across scales with an identical response–a response pattern commonly associated with careless responding [46]. This conservative screen flagged only 17 cases, and these cases were removed from subsequent data analysis. Furthermore, none of the remaining 1695 cases failed an instructed response item embedded in the trust in science scale (“please select disagree for this item”).

In order to conduct our analyses using regression and relative weights analysis, we chose to only include participants that chose male or female for the gender item, and either Donald Trump or Joe Biden for 2020 voting item. As the sample of most nonwhite subgroups were too small for meaningful comparisons, race was recoded as white or nonwhite to maintain the maximum number of participants to be included in the model. This allowed us to include these items as dichotomous predictors in our analyses. All other predictors maintained their ordinal/continuous scale. Intercorrelations and descriptive statistics are provided in Table 1.

**Table 1:** Intercorrelations between study variables.

	*M *	*SD *	1	2	3	4	5	6	7	8	9	10	11	12	13	14	15	16
1. Density	276	162	1															
2. Age	43.4	17.5	-.05*	1														
3. Education	3.6	1.4	.11**	.19**	1													
4. Female	.74	.44	.03	-.12**	-.08**	1												
5. Nonwhite	.29	.45	-.04	-.19**	-.07**	.06*	1											
6. Mild Case	.46	.50	.03	-.07**	.09**	.05	-.01	1										
7. Died Case	.15	.35	-.02	.02	.09**	.01	.13**	.26**	1									
8. Trump Vote	.51	.50	.00	.16**	-.06*	-.09**	-.39**	-.04	-.07**	1								
9. Trump Approval	.48	.50	-.02	.15**	-.08**	-.07*	-.35**	-.03	-.04	.94**	1							
10. Conservative	4.2	1.7	.03	.25**	-.02	-.07*	-.21**	-.02	-.06*	.64****	.61**	1						
11. COVID-19 Knowledge	9.6	1.7	.11****	.20**	.19**	.06*	-.27**	.08**	.01	-.08**	-.13**	-.09**	.63					
12. Need for Cognition	5.0	.99	.02	-.01	.17**	-.08**	-.02	.04	.03	-.02	-.02	-.03	.12**	.85				
13. Trust in Science	4.7	1.0	.04	.07**	.18**	-.04	-.06*	.03	.01	-.39**	-.35**	-.38**	.32**	.16**	.94			
14. Party Identification	4.1	2.3	.00	.19**	.00	-.07*	-.37**	-.03	-.09**	.81**	.76**	.67**	-.04	-.06*	-.31**	1		
15. Preventative Behaviors	3.4	.62	.06*	.16**	.07**	.09**	.07**	.01	.05*	-.23**	-.25**	-.18**	.33**	.10**	.31**	-.19**	.70	
16. Vaccine Intentions	.53	.50	.05*	.14**	.17**	-.16**	-.12**	.04	-.02	-.11**	-.10**	-.12**	.14**	.04	.36**	-.06*	.23**	1

Note. M = mean; SD = standard deviation. Values in the diagonal are internal consistency estimates where appropriate.

* p < .05

** p < .01.

To test our research question, we first conducted hierarchical multiple regression whereby our predictors were regressed on our three-item preventative behaviors’ scale. As the aforementioned research has substantiated correlations between many demographic variables and preventative behaviors, we entered density, age, race (white vs. nonwhite), gender, and education level in the first step of our model. All of these control variables, except education level, were statistically significant predictors of preventative behaviors and accounted for a substantial portion of variance (*R*^*2*^ = .052). Next, we entered in our remaining predictors to examine whether they demonstrated incremental validity. Indeed, the second step was incrementally predictive of preventative behaviors (*ΔR*^*2*^ = .139; see Table 2), and the full model predicted 19.1% of the variance in participants’ preventative behaviors (*F*_(14, 1179)_ = 19.88, *p* < .001, *R*^*2*^ = .191). Importantly, trust in science (*β* = .216), Trump’s approval rating (*β* = -.178), and knowledge scores (*β* = .158) were the three strongest predictors in our model. These results are robust and hold under a variety of alternative specifications, including iterative processes to select the regression coefficients.

**Table 2:** Multiple regression analyses predicting preventative behaviors.

	Step 1		Step 2	
	*β*	Full Model	*β*	Full Model
Density	.060*		.041	
Age	.154*		.153*	
Female	.086*		.085*	
Nonwhite	.162*		.135*	
Education	.053		-.030	
Knowledge			.158*	
Need for Cognition			.038	
Trust in Science			.216*	
GOP Identification			-.014	
Trump Vote			.091	
Trump Approval			-.178*	
Mild Case			-.002	
Died			.058*	
Ideology			-.029	
*R* ^*2*^		.052*		.191*
Adjusted *R*^*2*^		.048		.181
*F*		13.077*		19.876*
*ΔR* ^*2*^				.139*

*(*p* < .05).

Next, we used the same two-step model to predict vaccine intentions via logistic regression. In the first step, all of our control variables except for density were statistically significant predictors of vaccine intentions, with 59.2% of participants’ vaccine intentions correctly predicted by the model (see Table 3). In the second step, our model demonstrated a much higher percent of vaccine intentions correctly predicted (69.4%), and several variables showed significant predictive validity. In particular, trust in science, female, age, Trump vote, GOP identification, knowing someone with a mild case, and conservatism were all statistically significant predictors.

**Table 3:** Logistic regression analyses predicting vaccine intentions.

	Step 1				Step 2			
	*b*	odds ratio	Wald	Full Model	*b*	odds ratio	Wald	Full Model
Density	.001	1.001	2.444		.001	1.001	3.635	
Age	.010	1.010	7.202*		.015	1.015	13.018*	
Female	.763	2.145	27.308*		-.890	.411	30.717*	
Nonwhite	.290	1.336	3.902*		-.333	.717	3.396	
Education	.161	1.175	13.345*		.090	1.094	3.291	
Knowledge					-.064	.938	1.623	
Need for Cognition					-.030	.971	.181	
Trust in Science					.739	2.095	82.818*	
GOP Identification					.146	1.157	7.519*	
Trump Vote					-1.162	.313	7.796*	
Trump Approval					.637	1.891	2.695	
Mild Case					.339	1.404	6.125*	
Died					-.364	.695	3.595	
Ideology					-.111	.895	4.255*	
Nagelkerke *R*^*2*^				.079				.246
% Correctly Predicted				59.2%				69.4%

*(*p* < .05). Vaccine intentions: 1 = No, 2 = Yes.

Due to the expected multicollinearity among our predictors, we chose to conduct a relative weights analysis a priori, although we did not make predictions regarding the importance of specific predictors. Relative weights analysis (RWA) is a useful tool that can supplement regression analyses by calculating the unique contribution of each predictor in a regression equation (see [47] for a discussion). Results from the RWA substantiated COVID-19 knowledge (31.71%), trust in science (20.57%), and Trump approval (9.30%) as statistically significant and practically meaningful predictors of COVID-19 preventative behaviors (see Table 4). Additionally, age (12.77%) was also a powerful predictor as it predicted the third most variance in the model. For vaccine intentions, trust in science (48.41%) predicted nearly half of the predicted variance, followed by being female (15.15%), age (6.64%), and being conservative (5.18%). Interestingly, Trump vote was a significant predictor for preventative behaviors (5.46%) and vaccine intentions (4.84%).

**Table 4:** Relative importance as percentage of predicted variance by predictor.

	Preventative Behaviors	Vaccination Intentions
Density	1.00%	1.18%
Age	12.77%*	6.64%*
Education	0.52%	4.62%*
Female	3.69%*	15.15%*
Nonwhite	4.03%*	3.48%
Mild Case	0.10%	1.83%
Died Case	0.72%	1.14%
Trump Vote	5.46%*	4.84%*
Trump Approval	9.30%*	3.13%
Conservative	3.90%*	5.18%*
COVID-19 Knowledge	31.71%*	1.70%
Need for Cognition	2.37%*	0.31%
Trust in Science	20.57%*	48.41%*
Party Identification	3.86%*	2.39%
*R* ^*2*^	.223*	0.188*

* values which relative weights’ 95% confidence intervals did not include 0.

Many of the political variables in our model did predict meaningful variance in preventative behaviors, as shown in the RWA results. Specifically, 2020 voting behavior/intentions (5.46%), ideology (3.86%), and party identification (3.86%) were all statistically significant predictors despite having nonsignificant beta weights in our regression analyses.

## Discussion

The purpose of this study was to construct and test models of COVID-19 preventative behaviors and vaccine intentions, and to compare the relative importance of various predictors drawn from several scientific fields. In order to control the transmission of COVID-19, as well as other infectious disease pandemics, it is vitally important for public health officials to efficiently educate the community about the best public health practices. By understanding the variables that contribute to compliance to these recommended practices, public health officials can better tailor their outreach efforts to educate the community. Indeed, targeted messaging and education are likely to be instrumental in executing interventions that enhance compliance with medical guidelines and advice, particularly in a period saturated by mediums (e.g., the internet) in which misinformation is fluid and convincing [48].

Knowledge about COVID-19 proved to be a major contributor to an individual’s practice of preventative behaviors. An increase in general knowledge about the transmission, symptoms, and epidemiology of the virus contributes to an increase in the compliance to preventative behaviors. It is vitally important when suggesting preventative practices, that public health officials focus on educating the community about the virus and disease. An individual’s willingness to wear a mask will be aided by the understanding of the mechanism of airborne transmission of the virus. Public health officials must aim to provide education about the facts and science surrounding SARS-CoV2 and COVID-19 in conjunction with their recommendations of preventative behaviors. This combination of education about COVID-19 with recommendation of safe practices will improve the likelihood of compliance within the community. It is important to note that there is a relationship between COVID-19 knowledge and race and education. There is clear evidence that minorities are at greater risk from COVID-19 infection and death. This can be due to disparities in healthcare access, housing, education, and discrimination [3]. Our findings show that knowledge scores were lower in nonwhite respondents. The decrease in COVID-19 knowledge only exacerbates the already existing health disparities in minorities. It is vitally important that minority groups are reached with educational outreach programs and provided access to health care and vaccination.

Similar to previous research, our findings indicate gender, race, and age are significant contributors to the practice of preventative behaviors. Older, Non-white, and female respondents were more likely to practice preventative behaviors. In terms of gender and race differences, efforts to increase compliance in these groups could include the use of respected leaders in the community that may have a better ability to gain the trust of their community and relay important public health messaging. Also reaching younger age groups with public health messaging may be improved through the use of social media and community influencers.

Interestingly, while gender and race were important predictors in both the regression and RWA analyses, the RWA found them to hold slightly lower predictive power compared to their standardized beta weights in the regression model. It is possible that the value of these demographic variables was more prominent in a regression model where collinearity concerns could not be fully addressed. In the RWA, the overlap of these variables with other predictors (e.g., ideology, voting behavior, and Trump approval) was accounted for and their unique contribution may have been overestimated in the regression analyses.

Also of interest, though we found females and nonwhites to have significantly higher preventative behavior, we also found them significantly less likely to report vaccine willingness. One potential explanation for these disparate trends in gender, is that females tend to take be less prone to risk-taking behavior in general [49]. Though taking a vaccine may not seem as risk acceptant as going without one, recall that the survey was fielded prior to FDA approval of any vaccines, which did not occur until December 11, 2020. So, respondents are asked only about hypothetical vaccines that are hypothetically approved, which may affect attitudes towards the vaccine. Thus, perhaps less risk-acceptant respondents would be willing to assent to such a hypothetical healthcare decision, while at the same time being more likely to be engaged in preventative behaviors.

Indeed, we probed self-reported rationales for vaccine hesitant respondents, and our results are consistent with pre-approval risk aversion among females. Subjects were given a variety of options to explain their vaccine hesitancy, including: prior infection, lack of faith in vaccine efficacy, lack of faith in vaccine safety, intentional harm from vaccines, lack of concern over COVID, and a rushed approval process. The modal outcome respondents selected was this concern over a “rushed approval process,” and this option was disproportionately selected by women (46% of vaccine-hesitant women vs. 38% of vaccine-hesitant men). Thus, it is possible the gender gap in vaccine hesitancy narrows upon evidence of vaccine safety and efficacy once approved.

As to race, there is prior evidence that African Americans have less positive attitudes about the seasonal flu vaccine [50] than whites. In addition, both African Americans and Hispanics have also been found to be more hesitant about the COVID-19 vaccine than whites [51, 52]. Some researchers attribute these differences to a variety of factors including greater concerns about safety/efficacy, already being infected with COVID-19, or lack of financial or health insurance means [51]. In addition, there is a known lack of trust of government and healthcare among African Americans and other minorities due to various historical events like the Tuskegee syphilis study [3]. In our own survey, there were not significant differences as to the justifications selected by nonwhites as opposed to whites. Thus, it appears a multitude of reasons does indeed drive the racial gap in vaccine hesitancy.

The role of age as a predictor (which is also known to correlate with ideology, voting behavior, and Trump approval) was not differentiated between analyses, as age was one of the top four strongest predictors in either analysis. Trust in science has also proven to be a significant predictor of preventative behaviors and vaccine intentions. Research has found that trust in science relates to COVID-19 behaviors in general [53], but more narrow investigations have begun to emerge that suggests that the effect of trust in science may be affected by individual differences, such as political identity. For example, a recent set of studies by Koetke *et al*. [54] found that the effect of political ideology on social distancing was moderated by trust in science, whereby conservative participants were more likely to social distance when they also reported higher trust in science. Thus, there appears to be interactive effects between political beliefs/affiliations and scientific trust that predict incremental variance in COVID-19 related behaviors, such as social distancing. Our work complements these studies by substantiating a strong, positive, and unique effect of trust in science beyond the related variables of political ideology, party identification, voting behavior, Trump approval, gender, race, age, and COVID-19 knowledge.

It is important to note that in our study, trust in science, political ideology, party identification, Trump approval, and 2020 voting intentions/behavior all accounted for statistically significant proportions of variance in COVID-19 preventative behaviors, however, some research has suggested that the effects of political ideology on individuals’ approval and subsequent adherence to preventative behaviors are fully mediated by trust in science [55]. While our study did not measure approval of prevention guidelines for behavior, our findings of direct effects for each of these variables suggests that, at least in South Carolina and possibly the United States more broadly, the pathways from individual political beliefs and identity constructs could flow in ways that are not subsumed by their influence on trust in science. Further, trust in science accounted for nearly half of all the variance predicted by our model in vaccine intentions, controlling for all other variables in our model, although Trump vote and ideology were also statistically significant predictors. While we were not able to dive further into the incredible impact that trust in science had on vaccine intentions, it is likely that the uncertainty and hypothetical nature of potential vaccine approval, combined with the extreme accelerated timeline of vaccine development, caused participants to draw more from their general trust in science and scientists when reflecting on their intentions to receive an approved vaccine.

Interestingly, COVID-19 knowledge was a strong, positive predictor of preventative behaviors but not vaccine intentions. This discrepancy may be both intuitive and contradictory. For instance, it seems reasonable that an individual who is knowledgeable regarding the symptoms, spread, and nature of COVID-19 would be more likely to conform to common prevention guidelines, but may not be more likely to intend to receive a vaccine. This could be due to the proximal nature of prevention behaviors compared to the more distal intention to receive a not-yet approved vaccine. On the other hand, one may expect that an individual who understands COVID-19 more fully would also understand, and perhaps appreciate, the utility of vaccinations in containing and potentially eliminating COVID-19. We believe this discrepancy is one that deserves more attention and should be addressed in a more narrow explanatory model. Need for cognition was not significant in either regression model, although it explained a significant but small portion of variance in preventative behaviors in the RWA. It is possible that a more complex model of COVID-19 knowledge, trust in science, and need for cognition may shed light on possible interactions or boundary conditions in their intersection with preventative behaviors and vaccine intentions.

We found here that political beliefs and identity significantly contributed to predicting the practice of preventative behaviors. Of the four political variables we chose (Trump approval, partisan identity, 2020 vote, and ideology), Trump approval was the strongest predictor of preventative behavior. No other political variables are significant with all variables included in the model.

The findings of the vaccine willingness model and the political variables are more muddled. All four variables are weakly and negatively correlated with vaccine willingness when paired in bivariate correlations. However, the full logit model reveals that while 2020 Trump voters are *less* willing to take the vaccine, relatively Republican-identifiers are significantly *more* likely to take the vaccine, when included alongside other predictors in our regression model. It is possible this finding is the product of multicollinearity between the political variables making the individual logit weights less stable. However, an examination of the descriptive data suggests this anomaly may be due in part to the fact that less partisan respondents (pure Independents and GOP-leaners) are the least likely groups to express vaccine willingness, as compared to strong partisans (either Democrats or Republicans).

In total, the political results suggest a Trump-specific effect with some form of support for the former President (current President at the time the survey was taken) being uniquely related to both less preventative behavior and less willingness to take the vaccine. For example, Trump vote was a significant predictor of both outcomes in our RWA analyses, where it’s overlap with other political variables was accounted for. Whether this is due to Trump’s lack of seriousness about the virus in his own communication, his supporters downplaying the virus in order to avoid facing a potentially damaging election issue, or other unidentified confounders related to Trump support is beyond the scope of this study. Still, these results do support a political and Trump-centered element to the publics’ COVID behavior that merits further investigation into the exact causal mechanism.

Due the online delivery of the survey, one limitation of this study is that only those with internet access could respond. An additional limitation is that while respondents were asked about their preventative behaviors, this survey does not address the accuracy of the practice of the behavior (i.e., wearing a mask correctly). While our sample offered a powerful representation of South Carolina residents, the extent to which our findings generalize to other areas of the country or other countries entirely is uncertain. Additionally, subjects had to self-select in to participate, whereby those choosing not to participate could have differed from those that did in some meaningful way. It also possible that our transformation of some variables into dichotomies may have artificially constrained their variance, lowering our power to detect effects. Nevertheless, we believe our replication of many previous empirical findings by use of different methodology and analytic tools suggests that there may be some generalizability.

In conclusion, our two models of antecedents predicted substantial proportions of variance in COVID-19 preventative behaviors and vaccine intentions. When we controlled for shared variance using RWA, results demonstrated that psychological (e.g., trust in science, COVID-19 knowledge), political science (e.g., Trump approval, voting behavior), and demographics variables (e.g., age, gender) were important independent predictors. Further, we believe that when feasible, the simultaneous modeling of constructs from various approaches may provide a more nuanced perspective moving forward as piecemeal research, which is informative and necessary in early stages of research program development, is extended toward explanatory models of infectious disease phenomena. Overall, the results of this study provide valuable information that can be used to improve the efficacy of public health messaging during the COVID-19 pandemic.

## Supporting information

S1 Data (XLSX)
